# Neurological Manifestations of Connective Tissue Disorders

**DOI:** 10.7759/cureus.47108

**Published:** 2023-10-16

**Authors:** Riddhi S Poshattiwar, Sourya Acharya, Samarth Shukla, Sunil Kumar

**Affiliations:** 1 Department of Medicine, Jawaharlal Nehru Medical College, Datta Meghe Institute of Higher Education and Research, Wardha, IND; 2 Department of Pathology, Jawaharlal Nehru Medical College, Datta Meghe Institute of Higher Education and Research, Wardha, IND

**Keywords:** headache, seizures, neuropsychiatric disorders, cranial nerve palsies, neuropathies, cognitive dysfunction, neurological manifestations, connective tissue disorder

## Abstract

Connective tissue disorders (CTD) are a group of disorders affecting the connective tissues. Usually the musculoskeletal and the vascular system is impacted. Along with these systems, the nervous system is also involved in CTD, which leads to various neurological manifestations. The pathophysiology of neurological complications of CTD is caused by various factors and is complicated. Disturbed immune complexes, chronic inflammation, and autoimmunity in which the body attacks its cells are considered to be responsible for the neurological complications of CTD. Additionally, the vascular symptoms that lead to decreased blood flow to the brain are also responsible for the neurological manifestations of CTD in diseases like systemic lupus erythematosus (SLE). In SLE, vessel wall integrity is compromised, which may lead to decreased blood flow leading to neurological complications. CTD can manifest a variety of neurological complications. These neurological complications can be classified into symptoms affecting the peripheral nervous system, central nervous system, and the autonomic nervous system. Some of the common neurological complications of CTD are headaches, seizures, ataxia, neuropathies leading to cranial nerve palsies, myelopathies, tremors, encephalitis, and cerebral infarction. Cranial nerve palsies can disturb sensations, vision, hearing, and mastication. Neuropsychiatric symptoms are also commonly observed in CTD. Cognitive dysfunction can be caused due to neuropsychiatric problems. Some of the cognitive dysfunctions are lack of concentration, memory loss, confusion, and coma. In this review, we will address various neurological manifestations of CTD.

## Introduction and background

The term connective tissue disorders (CTD) refers to a collection of multisystemic, chronic inflammatory autoimmune diseases that are brought on by an immune system reaction involving an antibody or T-cells that target self-antigens known as autoantibodies and ultimately results in tissue destruction and organ failure. These are acquired disorders. An overt CTD results from a combination of environmental stressors and the immune system's genetic makeup [1]. Clinicians can diagnose CTD by detecting the specific autoantibodies of the disease [2].

Autoantibodies found in cases of systemic lupus erythematosus (SLE) include anti-dsDNA autoantibodies, anti-nucleosome autoantibodies, anti-Sm autoantibodies, anti-histone autoantibodies, anti-Ro, and anti-La autoantibodies [2]. In rheumatoid arthritis (RA), autoantibodies found are rheumatoid factor (Rf) and anti-citrullinated protein antibody (ACPA) [3]. Anti-Scl70/ DNA topoisomerase 1, anti-centromere, anti-RNA polymerase 3, anti-U3-RNP, and anti-Pm/Scl are the autoantibodies detected in patients with systemic sclerosis [4]. In Sjögren’s syndrome (SS), anti-Ro52, anti-Ro60, and anti-La/SSb autoantibodies are detected [5]. Anti-phosphatidylserine-prothrombin complex (anti-PSPT) antibodies and anti-moesin antibodies are present in patients with polyarteritis nodosa (PAN) [6]. In granulomatosis with polyangiitis, the presence of anti-nuclear cytoplasmic antibodies (ANCA) can be seen [7]. CTDs are more common in women [8]. CTD can present as articular and extra-articular manifestations.

In this review article, we will explore how various CTDs present neurologically. Central nervous system (CNS) and peripheral nervous system (PNS) involvement may be a result of neurological problems with CTD [1]. We will focus on discussing the key autoimmune multisystemic disorders such as SLE, RA, scleroderma, SS, Churg-Strauss syndrome (CSS), Dermatomyositis, PAN, and granulomatosis with polyangiitis. These conditions are notable for their neurological complications compared to other CTDs.

## Review

Methodology

**Figure 1:**
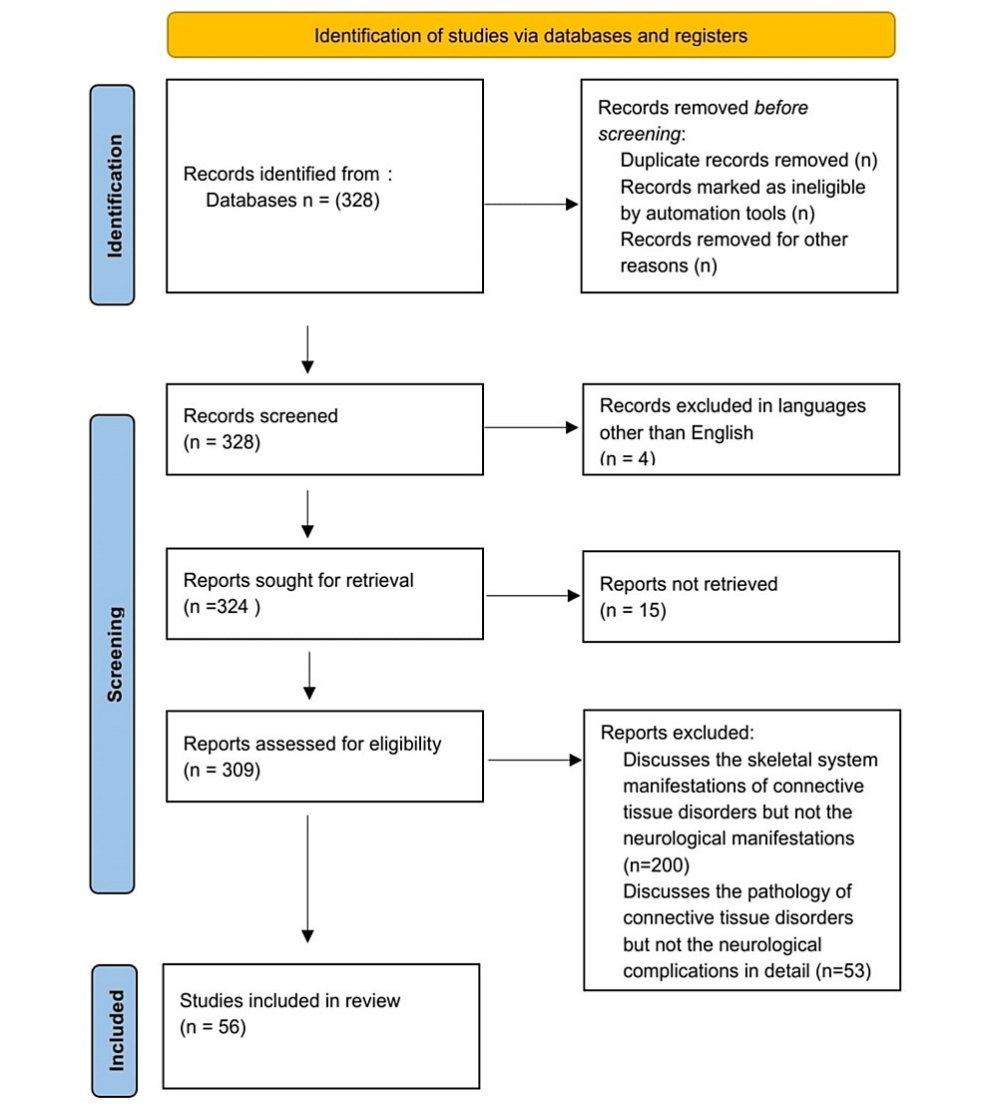
PRISMA flow diagram for the process of selection PRISMA: Preferred Reporting Items for Systematic Reviews and Meta-Analyses

A detailed search was done on PubMed using key terms, such as “neurological symptoms,” “connective tissue disorders,” “extra-skeletal symptoms,” “Systemic lupus erythematosus,” “Rheumatoid arthritis,” “Scleroderma,” “Sjogren’s syndrome,” “Churg-Strauss Syndrome,” “ Dermatomyositis,” “Polyarteritis nodosa,” “Granulomatosis with Polyangitis," were used interchangeably and in combination. All the articles that discuss the neurological symptoms of CTDs were included. Articles that were not retrievable or discussed either the skeletal system symptoms or the pathology of the CTDs were excluded. Out of the 328 articles identified, 56 were deemed relevant and were included. These articles were included based on their focus on the neurological complications of CTD, as opposed to others that primarily discussed either CTD manifestations related to the skeletal system or the general pathology of CTD. Figure 1 depicts the Preferred Reporting Items for Systematic Reviews and Meta-Analyses (PRISMA) flow chart for the literature search.

SLE

SLE is a chronic, systemic autoimmune disease with a variable course [9]. In SLE, the CNS is damaged either directly by damaging the neurons and arteries of the brain or indirectly by the processes that are responsible for the production of immune complexes and their accumulation [10]. Neuropsychiatric SLE (NP-SLE) is characterized by the involvement of the CNS and peripheral nervous system (PNS) [11]. The most frequent neuropsychiatric symptom is headache. Standard analgesic therapy is ineffective for treating a lupus headache [12]. Diagnosis of psychosis is also frequently made in NP-SLE patients [13]. Patients with NP-SLE experience cognitive impairment, which can include issues with concentration, frank confusion, and coma [14]. These individuals frequently present with seizures. The frequency of generalized seizures is more than that of focal seizures [15]. NP-SLE patients sometimes have movement abnormalities as well. Chorea, cerebellar ataxia, hemiballismus, tremors, and movements that resemble Parkinsonian symptoms are all examples of movement disorders [16]. The most widespread movement problem is chorea, which is more prevalent in pediatric SLE than in SLE in adults [17]. Optic neuropathy is a well-known neuro-ocular symptom that appears in people with SLE [18]. 

RA

RA, a chronic condition, is distinguished by the presence of autoantibodies, persistent synovitis, and widespread inflammation. Notably, neuropsychiatric signs, such as depression, cognitive deterioration, abnormal behavioral patterns, compression of the spinal cord, and peripheral neuropathy, are notably common in RA [19]. The likely causes include generalized systemic inflammatory mechanisms, nerve compression brought on by degenerative changes in the bones and joints, negative drug side effects, and accelerated atherosclerosis brought on by both systemic inflammation and autoantibodies [19]. A variety of neurological conditions can develop as a result of RA, including complications caused by rheumatoid vasculitis which include ischemic neuropathies, entrapment neuropathies, cervical spine complications such as rheumatoid meningitis, atlantoaxial subluxation, basilar invagination, and, in rare cases, vasculitis or amyloidosis [20].

Neurological manifestations arise from inflammation occurring within the atlantooccipital and atlantoaxial joints, mirroring the inflammatory processes observed in peripheral joints, ultimately leading to the formation of pannus and the degradation of adjacent bones [21]. Through more common subluxations, this inflammation can cause indirect compression of numerous structures such as the spinal cord, vertebral arteries, cranial nerves, and nerve roots, or in rare circumstances, it can lead to direct compression through inflammatory tissues. The type of compression depends on the particular joint that is being compressed [21]. In contrast, compression neuropathies arise due to the pressure applied on peripheral nerves, with the median nerve being a frequently affected site leading to conditions like carpal tunnel syndrome. This compression can arise from inflamed joint synovia or the inflammation-induced swelling of adjacent tendons, a condition referred to as tenosynovitis [22]. Ischemic neuropathies triggered by rheumatoid vasculitis predominantly present in several various forms such as sensory neuropathy, motor neuropathy, multifocal neuropathy, and distal symmetrical sensory neuropathy. These manifestations arise due to vasculitic alterations impacting the vasa nervosa, the small blood vessels that supply nerves [21].

Scleroderma 

Scleroderma is a chronic fibrosing autoimmune illness that affects the skin and numerous other organs. It is also known as systemic sclerosis [23]. The intricate origin of the disease is attributed to the initial vascular injury, inflammatory infiltration, and fibrosis [23]. Due to genetic predisposition, an imbalance in the innate and acquired immune systems leads to the release of numerous immunological messengers and autoantibodies. This leads to the activation of fibroblasts, which subsequently leads to the formation of myofibroblasts and rigid connective tissue [23-24]. The nervous system's involvement can develop years after the initial symptoms of the disease appear and is usually unrelated to skin activity [25]. In 16% of the patients, the neurological symptoms precede the appearance of cutaneous symptoms [26]. A study of 54 patients found that 35% of patients overall and 11% of patients at presentation had localized neurological abnormalities [26].

According to a review of 54 individuals with linear scleroderma en coup de sabre and progressive facial hemiatrophy, 73% of patients experienced seizures, 33% of which were drug-resistant [27]. Tonic-clonic, absence seizures, status epilepticus, and complex partial seizures have all been observed more frequently [28]. Although cutaneous involvement may be the source of extraocular movement dysfunction and facial palsy, masticatory spasms and trigeminal neuralgia are considered to represent the major neurologic involvement [29-30]. Headache is referred by about 35% of linear scleroderma en coup de sabre patients, and it frequently coexists with other neurologic complaints [26]. Some research studies have examined the subtypes of headaches, but migraines and their imitators seem to detrimentally be more common [31]. 

SS

SS is a CTD that affects the structural and operational integrity of exocrine glands. This affliction transcends boundaries, potentially influencing the functionality of various internal organ systems [32]. Notably, distressing neurological conditions are observed as additional manifestations of this syndrome [32]. Existing literature suggests that a considerable portion, ranging from 8.7% to 50%, of SS patients concurrently experience neurological indications [33]. The precise mechanisms by which SS inflicts damage upon the neurological system remain elusive at the pathogenetic level. Notably, T lymphocytes and dendritic cells are believed to wield substantial influence due to their proficiency in generating cytokines, thereby culminating in vasculitis and subsequent impairment of the dorsal root ganglia, prompted by inflammatory infiltration. Furthermore, investigations are underway to identify specific antibodies capable of eliciting responses against antigens within the neurological framework [34]. Numerous signs that impact the CNS include cognitive impairments, aseptic meningitis, non-infectious seizures, ongoing headaches, transverse myelitis, optic nerve inflammation, and extensive encephalopathy [35]. Among these manifestations, cognitive impairments stand as a common occurrence in individuals with SS [35]. Meningitis emerges as a relatively frequent consequential impact of SS. It is a type of aseptic meningitis that is not caused by any pathogens [36]. Limited occurrences of possible distinctive neurological symptoms include seizures, cranial nerve dysfunction, or abnormalities in the cerebellum [35].

Extensive documentation exists regarding the occurrence of bilateral retrobulbar optic neuritis in individuals afflicted by SS. On occasions, this ocular complication has even manifested as the initial symptom, inducing blindness in both eyes [37]. The etiology behind optic neuritis within SS patients is believed to stem from a convergence of demyelination processes and the incitement of ischemic vasculitis [37]. In the context of SS, the spinal cord is most frequently targeted by episodes of acute transverse myelitis. The resulting clinical indications vary, encompassing conditions such as partial or complete paralysis of the limbs, impairments in sphincter control, disturbances in proprioceptive perception, and instances of Brown-Séquard syndrome, with the specific manifestations contingent upon the precise location of the focal lesion [32]. Various forms of neuropathies stand as the principal indicators of the involvement of the peripheral nervous system in SS [38]. Based on current knowledge, the prevalence of neuropathy within the SS population ranges from 2% to 60%, encapsulating a diverse spectrum of individuals [39]. The prevalent neuropathic manifestation in individuals with SS is distal axonal sensory polyneuropathy, which unfolds with a persistent trajectory marked by escalating symptomatology [40]. This condition presents as sensory impairments localized to the extremities of the lower limbs, giving rise to symmetrical paraesthesias. Often, these paraesthesias coincide with a sensation of burning discomfort in the feet. The severity of these symptoms is notably heightened within the lower limb regions [40]. Some individuals experiencing distal axonal sensory polyneuropathy also report motor nerve fiber involvement. As the ailment progresses, a discernible weakening of the peripheral muscles sets in, predominantly targeting the distal musculature of the limbs. The initial manifestation often revolves around the extensor muscles of the feet [40]. 

Within the field of SS, the occurrence of chronic inflammatory demyelinating polyneuropathy (CIDP) is infrequent. This condition follows a gradual progression, unfolding over a span of no less than eight weeks, and manifests as a bilateral and evenly distributed weakening of the muscles located in both extremities. Alongside this muscular decline, sensory disturbances and deep tendon reflexes are absent or diminished [41]. In the context of multiple mononeuropathy, a simultaneous or consecutive occurrence of unequal impairment occurs in a minimum of two nerves, without any anatomical continuity between them. Observable symptoms emerge in regions innervated by these compromised nerves [32]. Multiple nerves can experience overlapping multifocal lesions, resulting in occurrences of muscular weakness and unusual sensations similar to those found in polyneuropathy [32]. The key factor influencing the neuropathic variant being studied arises from inflammation affecting the vascular networks supplying the nerve bundles, ultimately resulting in nerve tissue ischemia [42]. From a clinical perspective, an area controlled by distinct nerve pathways experiences significant impairments affecting both sensory and motor functions. The onset of this condition typically begins with an acute or subacute episode lasting for days to weeks, characterized by simultaneous discomfort concentrated in the proximal area of the affected limb. Additionally, a troubling abnormal sensation emerges, corresponding to the sensory nerve distribution. Conversely, the initial phase may present without prominent pain but rather a reduction in strength in the dominant limb [32].

Sensory ganglionopathy is another manifestation associated with SS. The main clinical feature involves an unsteady gait characterized by a broad support base. People with sensory neuropathy face significant mobility limitations due to the instability of their walking, often requiring the use of a wheelchair for movement. Additional characteristics, other than the mentioned gait instability, include impairment in vibratory perception and adequate muscular strength accompanied by the lack of deep tendon reflexes [43]. Instances of pseudoathetoid motions, characterized by erratic and uncontrollable limb movements, have been documented in select cases, thereby giving rise to the prospect of extrapyramidal symptoms. This concern typically deteriorates over a span of several weeks, displaying a subacute progression pattern [44]. The presence of symmetrical symptoms is a common occurrence in sensory neuropathy. However, this is not a universal rule. Unlike multiple mononeuropathy or distal sensory neuropathy, the symptoms of sensory neuropathy can extend beyond mere symmetry. They can encompass the trunk region in addition to the entirety of the limb, presenting a broader scope of involvement [43]. Among cranial neuropathies observed in individuals with SS, sensory impairment of the trigeminal nerve (V) stands as the most commonly documented type. This manifestation predominantly targets the primary branch, known as the maxillary nerve, and tends to manifest unilaterally. Additional complications such as hearing loss, facial nerve neuropathy, disruptions in the vestibulocochlear nerve (VIII), and vestibular symptoms have all been associated with patients afflicted by SS [37].

In some cases, individuals affected by SS encounter irregularities within the motor neurons, which are alternately known as anterior horn cell disorders. In most of the recorded cases, observable signs such as muscular weakness, wastage, and fasciculations (spontaneous and irregular muscle fiber contractions) predominantly manifested within the peripheral segments of the limbs. Notably, no other indicative symptoms were present to further corroborate impairment of the upper motor neurons [45]. A solitary case of hypokalemic periodic paralysis occurring within the context of SS has been documented. This case was additionally associated with challenges related to renal tubular acidosis. In another patient, a deficiency in vitamin D was identified. Notably, even though the signs of dryness were little or nonexistent, the Schirmer test was positive [46]. Orthostatic hypotension, cardiac arrhythmias, irregularities in gastrointestinal motility, bladder dysfunction, disruptions in secretory activities, and instances of Adie's syndrome are a few of the signs that the ANS is at work. Numerous organ-specific methods are used to control how autonomic systems are involved in SS [47]. The majority of symptoms emerge due to the antagonism of type 3 muscarinic receptors. Potential triggers for the development of dysautonomia encompass the degeneration of autonomic nerve fibers within the exocrine gland cells, cytokines that obstruct cholinergic neurotransmission, and the inflammatory infiltration of T lymphocytes near the nerves and root ganglia of the ANS [47].

CSS

CSS, also known as eosinophilic granulomatosis with polyangiitis, is a form of widespread and severe vasculitis characterized by the inflammation of blood vessels and the presence of granulomas outside the vessels [48]. The vasculitic manifestations of CSS, such as glomerulonephritis, are likely influenced by antineutrophil cytoplasmic autoantibodies. On the other hand, eosinophil infiltration into tissues and its associated cytotoxic effects are believed to contribute to cardiomyopathy. CSS is frequently found in individuals with asthma and elevated eosinophil levels [49]. Aside from affecting various systems, the disease can lead to various neurological complications. These may manifest as peripheral neuropathy, multiple mononeuropathy, distal symmetrical polyneuropathy, and occasionally asymmetrical polyneuropathy. There have been reported cases of bilateral trigeminal neuropathy, radiculopathies, and ischemic optic neuropathy associated with this condition [50]. 

Dermatomyositis

Dermatomyositis is a complex and chronic autoimmune disorder with a range of contributing factors. It is known for its distinctive skin alterations and its impact on various organ systems, which can include muscles, blood vessels, joints, the esophagus, and the lungs [51]. In the muscular system, this disease presents as inflammation in skeletal muscles, leading to progressive symmetrical proximal myopathy, alongside classic skin-related symptoms [52]. Additionally, dermatomyositis may occasionally present with complications affecting the nervous system. These neurological complications can encompass polyneuropathy, carpal tunnel syndrome, trigeminal sensory neuropathy, ulnar sensory neuropathy, and involvement of the brachial plexus [53].

PAN

Another connective tissue condition with neurological symptoms is PAN. Medium-sized arteries are affected by the necrotizing vasculitis known as PAN [54]. The CNS and PNS symptoms are the two categories of neurological manifestations. The vasa nervorum enters the nerve and divides into intricate, anastomosing microvascular networks that largely run within the epineurium, the connective tissue that covers peripheral nerves and fills the interfascicular gap [54]. Necrotizing arteritis of the epineurium's vasculature is brought on by PAN-associated vasculitides, which also infrequently affect the perineurial and endoneurial vessels. Seizures, cerebral infarction, encephalitis, and peripheral neuropathies are among the symptoms [54].

Granulomatosis with polyangiitis 

A small-vessel vasculitis that is granulomatous and necrotizing is known as granulomatosis with polyangiitis. It was earlier known as Wegner’s granulomatosis. This disease is associated with the presence of ANCA, which suggests an autoimmune mechanism, but the exact cause of this uncommon illness is unknown [55]. The pituitary gland, cranial nerves, meninges, orbit, optic chiasma, and optic nerve may be one or more of the first tissues to be invaded by granulomatous tissue. The second and the least frequent mechanism involves the parietal bone and granulomatous intracerebral lesions of the brain. Third, brain or spinal cord vessels may be affected by vasculitis [55]. Clinically speaking, chronic hypertrophic pachymenitis (CHP) is the manifestation of Wegner's granulomatosis. The majority of the time, granulomatosis with polyangiitis-related cerebral CHP is the root cause of persistent, severe headaches that are often analgesic-resistant [55]. Oculomotor, trigeminal, abducens, facial, and vestibulocochlear nerves are the most commonly affected by the palsies caused by CHP. Granulomatosis with polyangiitis is also associated with posterior lobe insufficiency, a condition that results in central diabetes insipidus [56]. Meningitis, encephalopathy, seizures, and limb palsy are further symptoms [55].

Table 1 summarizes the neurological manifestations of CTD.

**Table 1:** Neurological manifestations of connective tissue disorders Credit: Riddhi Poshattiwar

Connective tissue disorders	Neurological manifestations
Systemic lupus erythematosus	Headache, cognitive dysfunction, chorea, cerebellar ataxia, hemiballismus, tremors
Rheumatoid arthritis	Compression of spinal cord, neuropathies, mononeuritis, cognitive dysfunction, depression
Scleroderma	Seizures, facial nerve palsy, trigeminal neuralgia, headache
Sjögren’s syndrome	Cognitive dysfunction, meningitis, seizures, headache, transverse myelitis, encephalopathy, vasculitis, neuropathies, trigeminal nerve palsy, optic neuritis
Churg-Strauss syndrome	multiple mononeuropathy, distal symmetrical polyneuropathy, asymmetrical poly neuropathy, bilateral trigeminal neuropathy, radiculopathies, ischemic optic neuropathy
Dermatomyositis	polyneuropathy, carpal tunnel syndrome, trigeminal sensory neuropathy, ulnar sensory neuropathy, brachial plexus abnormalities
Polyarteritis nodosa	Seizures, cerebral infarction, encephalitis, neuropathies
Granulomatosis with polyangiitis	Chronic hypertrophic pachymeningitis, encephalitis, seizures

## Conclusions

CTD is a s set of autoimmune, chronic inflammatory diseases with both extra-articular and articular symptoms. Acquired CTD can also present with neurological complications. In SLE, the neurological complications include seizures, headaches, chorea, cerebellar ataxia, tremors, and cognitive dysfunction. Neuropathy, cognitive dysfunction, and compression of the spinal cord are some manifestations of RA. Facial nerve palsy, trigeminal neuralgia, and seizures are linked to scleroderma. SS frequently includes neurological problems ranging from paraesthesia to different neuropathies, meningitis, severe transverse myelitis, encephalopathy, headache, meningitis, trigeminal nerve palsy, and cognitive impairment. Multiple mononeuropathy, distal symmetrical polyneuropathy, occasionally asymmetrical polyneuropathy, trigeminal neuropathy, radiculopathies, and ischemic optic neuropathy are some neurological symptoms of CSS. Polyneuropathy, carpal tunnel syndrome, trigeminal sensory neuropathy, ulnar sensory neuropathy, and involvement of the brachial plexus are all symptoms of dermatomyositis. Another condition that has neurological symptoms is PAN. These include cerebral infarction, encephalitis, seizures, and neuropathies. CHP, encephalitis, and seizures are some neurological complications of granulomatosis with polyangiitis. 
